# The Switch From Trivalent to Bivalent Oral Poliovirus Vaccine in the South-East Asia Region

**DOI:** 10.1093/infdis/jiw602

**Published:** 2017-07-01

**Authors:** Sunil Bahl, Andreas Hasman, Abu Obeida Eltayeb, Douglas James Noble, Arun Thapa

**Affiliations:** 1 World Health Organization, Regional Office for South-East Asia, New Delhi, India;; 2 UNICEF, Regional Office for South Asia, Kathmandu, Nepal; and; 3 UNICEF, East Asia and Pacific Regional Office, Bangkok, Thailand

**Keywords:** switch, trivalent oral poliovirus vaccine, bivalent oral poliovirus vaccine, immunization

## Abstract

This analysis describes an innovative and successful approach to risk identification and mitigation in relation to the switch from trivalent to bivalent oral polio vaccine (OPV) in the 11 countries of the World Health Organization’s (WHO’s) South-East Asia Region (SEAR) in April 2016.

The strong commitment of governments and immunization professionals to polio eradication and an exemplary partnership between the WHO, United Nations Children’s Fund (UNICEF), and other partners and stakeholders in the region and globally were significant contributors to the success of the OPV switch in the SEAR. Robust national switch plans were developed and country-specific innovations were planned and implemented by the country teams. Close monitoring and tracking of the activities and milestones through dashboards and review meetings were undertaken at the regional level to ensure that implementation time lines were met, barriers identified, and solutions for overcoming challenges were discussed and implemented.

The SEAR was the first WHO Region globally to complete the switch and declare the successful withdrawal of trivalent OPV from all countries on 17 May 2016.

A number of activities implemented during the switch process are likely to contribute positively to existing immunization practices and to similar initiatives in the future. These activities include better vaccine supply chain management, improved mechanisms for disposal of vaccination-related waste materials, and a closer collaboration with drug regulators, vaccine manufacturers, and the private sector for immunization-related initiatives.

The SEAR of the WHO consists of 11 countries, 6 of which are also in the UNICEF Region for South Asia (ROSA): Bangladesh, Bhutan, India, Maldives, Nepal, and Sri Lanka; and 5 in the UNICEF East Asia and Pacific Region (EAPR): Democratic People’s Republic of Korea, Indonesia, Myanmar, Thailand, and Timor-Leste. The WHO’s SEAR has been free of wild poliovirus since 13 January 2011, but there is continued risk of importation and spread of wild poliovirus from infected countries. Countries also face the risk of paralysis due to the emergence of vaccine-derived polioviruses (VDPVs), because OPV contains live attenuated strains of poliovirus that can sometimes mutate and revert to virulent form [1]. VDPVs are primarily due to the type 2 component of OPV. An outbreak of circulating type-1 VDPV was reported in Indonesia in 2005 (46 cases), while outbreaks of type-2 circulating VDPVs were detected in Myanmar in 2007 (4 cases) and in 2015 (2 cases), and in India in 2009–2010 (18 cases). In addition, 30 noncirculating VDPVs have been reported in the region since 2003, most of them due to the type-2 strain. Very rarely, OPV can also cause vaccine-associated paralytic poliomyelitis (VAPP), estimated globally at 2–4 cases per million birth cohort per year [2].

As part of the efforts to reduce the risk of VDPV outbreaks and VAPP, in December 2012 countries in the region discussed and agreed on the Global Polio Eradication and Endgame Strategic Plan 2013–2018 (the “Endgame” Plan) [3]. The withdrawal of OPV in a phased manner, beginning with the type-2 component of the vaccine, and the introduction of inactivated poliovirus vaccine (IPV) was 1 of the 4 objectives of the strategic plan. The WHO’s Strategic Advisory Group of Experts recommended that the traditionally used trivalent OPV (tOPV) should be replaced by bivalent OPV (bOPV) in both routine immunization and supplementary immunization campaigns in April 2016 [4].

A switch from tOPV to bOPV was expected to lead to declining immunity against poliovirus type 2, which, in turn, could potentially increase the risk of new circulating VDPV (cVDPV) type-2 outbreaks postswitch. It was recommended, therefore, that IPV be introduced to mitigate the risks associated with the decline in type-2 immunity. It was also recommended that all type-2 poliovirus materials or potentially infectious materials should be destroyed or contained in accordance with the WHO’s Global Action Plan III [5].

It became clear that the switch would pose significant operational and communication challenges in South-East Asia. The region has a birth cohort of 37 million, the highest of any WHO Region, with vast networks for storage and delivery of vaccines to meet the needs of children. There is significant variation between (and in some cases, within) countries in terms of health infrastructure and capacity, strength of medical regulation, the role of the private sector, data and information systems, supply chain management, and policies and practices for the collection and destruction of redundant vaccine. The constrained IPV supplies globally, due to difficulties in scale-up of IPV manufacturing, led to delays in introduction in some countries of the Region, and to stock-outs in some that had already begun introduction.

To help mitigate the risk of outbreaks of poliovirus type-2 postswitch, supplementary immunization activities with tOPV were conducted in India, Indonesia, Myanmar, Nepal, and Timor-Leste in the months leading up to the switch to build background immunity to type-2 poliovirus. In addition, all SEAR countries, except Indonesia, introduced IPV in their expanded program on immunization (EPI) programs prior to the switch. Indonesia introduced IPV after the switch in July 2016 in order to use a domestically produced vaccine. IPV supplies were assured for the countries at higher risk of VDPV emergence in the region; namely, India, Indonesia, Myanmar, and Timor-Leste. To mitigate the risks associated with a constrained supply of IPV, 8 states of India adopted an alternative immunization schedule in April 2016, providing 2 fractional doses of IPV delivered intradermally instead of a single full dose. The alternative schedule was based on the WHO position paper [2] and a study carried out in Bangladesh in 2015 [6]. This approach was expected to help stretch available supplies and reduce overall costs. The region is currently assessing the operational challenges associated with this approach. In July 2016, Sri Lanka also moved from a schedule of a single full-dose of IPV to a 2-fractional-dose schedule.

## PHASES AND OUTCOMES

In response to the challenges and potential risks of the switch in the region, the key stakeholders adopted a 4-phased approach. In the first phase, region-specific critical milestones and significant risks were identified based on global recommendations and guidance, and a review of the national switch plans in the region took place. In the second phase, a regional monitoring process was established, involving the WHO and UNICEF regional offices, with an aim to track progress and identify barriers and challenges in the lead-up to the switch. In phase 3 (2 months prior to the switch), the regional offices of the WHO and UNICEF conducted a consultation meeting that involved all country stakeholders, with the aim to identify country-specific issues and corrective measures. Finally, a regional reporting process was created to summarize the information from in-country independent monitoring and validation of the switch. This final step was used to evaluate the 4-phased approach taken for implementation of the switch in SEAR.

### Global Guidance and Regional Planning

Initial recommendations and guidelines for the switch were finalized in a meeting of the global Immunization Systems Management Group (IMG) in Seattle, WA, in April 2015. The meeting identified the challenges and possible solutions associated with the switch regarding vaccine licensure, availability of bOPV, withdrawal and disposal of tOPV, and communication with health workers and caregivers. A regional work plan with activities and time lines was developed jointly by the WHO and UNICEF to support switch planning in all countries in the region.

As part of preparations for the development of national switch plans in the region, a “dry run” of the switch was conducted in India in April 2015. India and Indonesia participated in this dry run, the first of its kind in the world. The key objectives of the exercise included a field testing of the global guidelines and tools developed for the switch, and an engagement of the key decision makers and stakeholders for the switch at the national and state levels. The India dry run demonstrated that the switch was feasible. The exercise highlighted that once the rationale of the switch was clearly explained, clear operational plans with potential barriers and solutions could be developed by the stakeholders to implement the switch. The key lessons learned from the dry run included the need for high-level advocacy on the rationale of the switch, and clear directives to the states from the national level on vaccine inventories and stock management to kick-start the process. Also of note, existing accountability structures could be used to manage the switch process, and involvement of the drug regulator, vaccine manufacturers, and the private sector vaccine providers were critical for success.

A meeting of the SEAR Immunization Technical Advisory Group (SEAR-ITAG) in June 2015 marked the beginning of the process to develop detailed switch plans in each of the 11 countries. Based on the lessons learned from the dry run, a template was used to initiate the process of developing national switch plans. The template identified critical milestones for the national immunization programs in the lead-up to the switch and the significant risks to be addressed at the planning stage. The process to identify time lines and persons responsible for each task was initiated. 

The SEAR-ITAG recommended that detailed national switch plans, including budgets, be finalized by each country by September 2015 [7]. The expert body also emphasized the importance of effectively managing stock inventories and tOPV stocks to ensure that there were no stock-outs of tOPV prior to the switch and minimal residual tOPV stock after the switch. The need to initiate the process of licensure and procurement of bOPV and the identification of mechanisms for the recall and disposal of tOPV immediately after the switch was also highlighted.

### National Switch Plans and Regional Monitoring

Following the ITAG meeting, the process to develop national switch plans commenced in all SEAR countries based on the SEAR-ITAG template with regional support from the WHO and UNICEF. Stakeholder engagement events were organized at the national level in all SEAR countries between July and September 2015, and technical experts were mobilized to finalize plans. The template invited in-country reflection on the current situation of OPV supply and distribution, waste management, licensure processes for vaccines, communication and training needs, and resources available and required for the switch. The situational analysis also involved identifying particular challenges and risks to the switch within the national context. In addition to the technical support provided by regional and country office WHO and UNICEF staff, 6 international consultants were identified and trained specifically in switch planning and preparation, and supported the development of draft plans in the countries.

The national switch plans were finalized in October 2015 with guidance from the WHO and UNICEF partners. The plans described the intended processes and structures for the implementation of the switch. In every country, the switch plans established a national switch coordination committee to oversee and coordinate national and subnational activities, and a national switch validation committee was set up to confirm the removal of tOPV based on the findings of independent monitors. The national switch plans also outlined plans for: (1) the development of strategies for vaccine procurement and distribution in UNICEF-procuring countries or self-procuring countries, (2) the collection and destruction of vaccine, (3) training, (4) communication, (5) budget requirements, and (6) monitoring.

**Figure 1.**
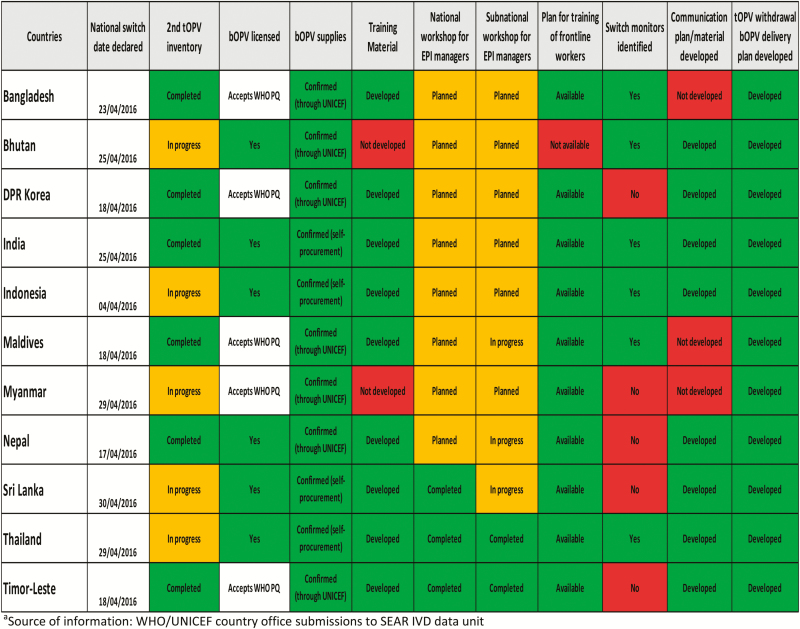
Regional status of preparedness for switch from tOPV to bOPV (as on 1 February 2016).^a^ Abbreviations: bOPV, bivalent oral polio vaccine; IVD, Immunization and Vaccine Development; SEAR, South-East Asia Region; tOPV, trivalent oral polio vaccine; UNICEF, United Nations Children’s Fund; WHO, World Health Organization.

Upon completion, the national switch plans were reviewed at the regional level by the WHO and UNICEF and, based on the findings, regional milestones and areas for particular attention were identified. Milestones were then integrated into a dashboard for completion by the WHO and UNICEF country teams, with input from national immunization programs and MoHs. The dashboard included a set of critical milestones to be achieved in each country during the process of switch planning and preparedness. More than 10 milestone indicators were tracked for each of the 11 SEAR countries using the dashboard (Figure 1). It was decided that UNICEF and WHO would independently complete the dashboard with a 4-week interval to ensure the completeness of data, and as a means of data cross-checking and verifying. Data on the various milestones were fed into a centralized master sheet, and a “traffic light signal” color scheme was used to indicate the status of progress in all countries on a variety of indicators.

The dashboard was frequently shared with country teams so that they could see their own progress and that of other countries. It helped the regional and country teams to focus on specific areas of underperformance and countries that needed specific technical support.

### Regional Consultation and Review

After the regional dashboard had been updated 3 times by the country teams between November 2015 and January 2016, a review meeting was held in February 2016 involving all countries. The regional teams presented the analysis of information from the dashboard to identify the main challenges facing the countries in the immediate lead-up to the switch. Overall, good progress had been made in the countries in terms of licensing and placement of orders for bOPV, and in the development of tOPV withdrawal and bOPV distribution plans. However, the second tOPV inventory had still not been carried out in 5 countries (Bhutan, Indonesia, Myanmar, Sri Lanka, and Thailand), and there was slow progress in identifying independent monitors for the switch in 5 countries (Democratic People’s Republic of Korea, Myanmar, Nepal, Sri Lanka, and Timor-Leste). Although risk communication and crisis communication strategies were found to have been completed or were in advanced stages of preparation in 8 countries, a need for follow-up was identified in Bangladesh, Maldives, and Myanmar. Training materials for switch-related activities were finalized in all countries except Bhutan and Myanmar. Bhutan also needed to complete a plan for training of frontline EPI staff.

All countries subsequently provided a progress update on switch preparatory activities and implementation with a focus on OPV inventories and supply adjustments, training of health workers, communication, vaccine distribution and withdrawal, and monitoring and validation. Following discussion of cross-cutting themes, each country team developed a detailed microplan for the activities to be conducted during the 2 months between the review meeting in February and the switch date, with actions and time lines in each thematic area of the switch process. The contents of the microplans confirmed areas of concern highlighted by the dashboard, and additional support was provided to countries as appropriate. Tracking of the national and subnational workshops and the finalization of the training/communication materials were undertaken to ensure that no delay in the switch occurred due to a delay in any of these critical activities. In coordination with the IMG, funding support was made available for Bangladesh, Democratic People’s Republic of Korea, India, Indonesia, Nepal, Maldives, Myanmar, and Timor-Leste. Guidance was also provided on the waste disposal mechanisms and the monitoring and validation process, including on the selection of monitors and monitoring methodology.

**Table 1.** IPV Introduction and OPV Switch in Countries of the SEAR

Country	Birth Cohort^a^	DTP3 Coverage 2015^b^	IPV Introduction		OPV switch	
			Schedule	Year	Switch Date	Date of Validation
Bangladesh	3228362	94	14 weeks	2015	23-Apr-16	7-May-16
Bhutan	12860	99	14 weeks	2015	25-Apr-16	9-May-16
DPR Korea	344435	96	14 weeks	2015	18-Apr-16	2-May-16
India	27420000	87	14 weeks	2015^c^	25-Apr-16	13-May-16
Indonesia	4893435	81	4 months	2016^d^	4-Apr-16	16-May-16
Maldives	7233	99	6 months	2015	18-Apr-16	12-May-16
Myanmar	1023892	75	4 months	2015	29-Apr-16	13-May-16
Nepal	614666	91	14 weeks	2014	17-Apr-16	11-May-16
Sri Lanka	334821	99	4 months	2015^e^	30-Apr-16	16-May-16
Thailand	675530	99	6 months	2015	29-Apr-16	13-May-16
Timor-Leste	44854	76	14 weeks	2016	18-Apr-16	23-Apr-16

Unpublished data compiled by Immunization and Vaccine Development Unit, WHO-SEAR. Abbreviations: DTP3, diphtheria-tetanus-pertussis; EPI, expanded program on immunization; IPV, inactivated polio vaccine; OPV, oral polio vaccine; SEAR, South-East Asia Region; UNICEF, United Nations Children’s Fund; WHO, World Health Organization.

^a^SEAR Annual EPI reporting form (AERF) 2015.

^b^WHO and UNICEF estimates of national immunization coverage, July 2016 revision.

^c^Phased introduction in India; 8 states of India administering fractional (1/5) dose of IPV intradermally at 6 weeks and 14 weeks since April 2016, and another 8 states to shift to this schedule from October 2016.

^d^Three doses of IPV being given in routine immunization in Yogyakarta Province since 2008.

^e^Sri Lanka shifted to a revised schedule of intradermal administration of fractional (1/5) dose of IPV at 2 months and 4 months since July 2016.

**Table 2.** Number and Percentage of Sites Monitored in the SEAR^a^

	National		Province		District		Government Delivery Points		Private Delivery Points	
	no.	%	no.	%	no.	%	no.	%	no.	%
Bangladesh	1	100	0	…	100	100	655	100	7	100
Bhutan	1	100	0	…	31	100	56	27	0	…
DPR Korea	1	100	11	100	210	100	4806	63	0	…
India	4	100	148	100	648	100	15385	59	5066	54
Indonesia	1	100	33	100	422	83	6366^b^	32	…	…
Maldives	1	100	0	…	19	100	155	100	1	100
Myanmar	1	100	22	100	302	92	613	34	0	…
Nepal	2	100	6	100	75	100	459	13	36	77
Sri Lanka	1	100	27	100	342	100	447	100	32	100
Thailand	1	100	967	100	0	…	4123^b^	35	…	…
Timor-Leste	1	100	13	100	0	…	88	100	7	100

Unpublished data compiled by Immunization and Vaccine Development Unit, WHO-SEAR. Abbreviations: SEAR, South-East Asia Region; WHO, World Health Organization.

^a^Source: Reports submitted by national validation committees of respective countries to the WHO–SEAR.

^b^Includes government and private delivery points (break-down of each not available).

### Regional Overview of Monitoring and Validation

At the regional level, a process was created for countries to report to the WHO on the outcome of the monitoring and evaluation processes. This was required to enable WHO Regional offices to report on the successful completion of the switch to the World Health Assembly. In addition, at the regional level, the reports from independent monitoring and validation in the countries were also used to evaluate the effectiveness of the 4-phased approach taken for the implementation of the switch in the SEAR. The national switch plans contained provisions for effective monitoring, reporting, and validation of tOPV withdrawal through the national switch validation committee based on the findings of independent monitors. Most countries broadened the scope of their National Certification Committee for Polio Eradication to take on the additional task of switch validation.

In each country, independent monitors were deployed to inspect vaccination stores and health centers that provide immunization services, and to report to the national validation committee. Monitors included staff from development partners and nongovernmental organizations, and government staff from various departments, including health, education, and social development. International monitors were deployed by the WHO (at least 1 to each country in the region) to support the monitoring process. A standardized monitoring template was used by all national and international monitors to collect information in order to assess the processes that had been put in place for the switch at the national and subnational levels, as well as record the outcomes of these processes. Mechanisms were also put in place for collection of daily reports from countries from switch day until the switch validation was certified by the country’s national switch validation committee. A master dashboard was designed at the SEAR Office to compile the monitoring data flowing in from the 11 countries and to review the progress in all countries.

The independent monitors’ findings were reviewed by the national switch validation committee. When the committee was satisfied that tOPV had been removed from the system at all levels, a recommendation was made to the MoH to sign the national validation report and forward a copy to the WHO SEAR Office. These reports were expected to include findings from global monitors that had been deployed in each country for this purpose.

By 17 May 2016, all countries of the Region had submitted their validation reports to the Regional Office for the SEAR, making this the first among all WHO regions to have completed the switch and its validation.

## DISCUSSION

The vaccination switch was an opportunity for countries to systematically engage with key issues relating to the delivery of routine immunizations. The process of switching vaccine entailed particular challenges relating to stock management of OPV at the national and subnational levels. Specifically, there were 3 objectives for the countries in the region: (1) sufficient stocks of tOPV at vaccination sites up to the time of the switch, (2) sufficient stocks of bOPV at vaccination sites from the switch date and onward, and (3) minimal surplus tOPV for collection and destruction following the switch. Most countries found that they did not have adequate and reliable information to adjust OPV stock at the subnational level, while at the national level there was a need for information to enable vaccine procurement and delivery adjustments to the states. Therefore, it was decided to take at least 2 inventories prior to the switch in every country and for immunization programs to report stock levels to a repository. Guidance and a template were issued in what turned out to be a major innovation. The repositories informed decision making on vaccine production, allocation, and procurement at national and global levels. The experience of periodic stock-taking for the switch is now contributing to the development of comprehensive data systems for immunization supply management in the region.

The vaccine manufacturers and stockists played a key role in ensuring that production and supplies of tOPV were regulated, to minimize surplus in the private sector in the run-up to the switch. A new area of collaboration opened up between the public health system and vaccine manufacturers and stockists. The drug regulators played a supportive role, not just in the licensure of bOPV but also in supporting the coordination with vaccine manufacturers and stockists and monitoring the switch process, especially in the private sector.

In preparation for the switch, countries found that policies and practices for the collection and/or destruction of vaccination waste and surplus vaccine were often inadequate or inconsistent. Guidance was provided to countries on options for the inactivation and disposal of all residual tOPV after the switch. Country-specific protocols for the disposal of tOPV were developed and implemented, depending on the quantities to be destroyed and facilities available in the country. These protocols have the potential to play a meaningful role in improving practices for disposal of vaccine and other immunization waste in countries of the region.

The development and use of a standardized monitoring template to collect information in order to assess the switch processes also provided important lessons for immunization programs across the region. The switch proved that it is feasible to collect daily reports from across countries to enable decision making at national and subnational levels. Although the model used for the switch is likely to be too resource intensive for routine immunization and stock management, the value of extensive monitoring using a standardized format early in the switch process became obvious. Immunization systems and development partners should consider the benefits of a standardized monitoring format when developing more cost-effective means for monitoring and reporting.

Training of health professionals such as vaccinators and cold chain managers was perhaps the least challenging aspect of the switch. The information on the rationale and processes of the switch communicated to the vast number of frontline health workers provides the confidence that public health messages can be disseminated effectively and in a timely manner through health systems, if required.

The private sector played a positive role in support of the switch. Medical and pediatric associations supported the dissemination of the importance of the switch and the role of their members, to ensure active participation of the private sector. A collaboration of the private sector in a public health program of this magnitude was unprecedented and opens the door for further collaborations between the public and private sectors with regard to future immunization and other public health interventions.

In the course of the switch, it also became clear that existing communication strategies for EPI were insufficiently developed in the countries. For this reason, switch-specific communication strategies had to be developed, informed by the communication guidance developed at the global level. Switch communication strategies used strategic approaches based on a thorough study of key audiences and the development of precise messages. Considering that the switch was a replacement of a vaccine and required no changes in caregivers’ practices and behaviors, it was decided to exclusively target proactive communication to health-care workers in order to prepare them for the switch. In most countries, communication to vaccinators and those involved in immunization logistics and supply focused on the role of the switch in polio eradication and, crucially, the concrete risks of using tOPV after the switch. However, although the primary focus was on health workers, due to the risk of coinciding adverse events that might be wrongfully attributed to the switch, and the possibility of a type-2 polio virus outbreak following the switch, countries also developed and tailored crisis communication strategies specifically for the general public. These strategies, focused on messages that clarified misconceptions, emphasized the reason for the switch and reiterated the importance of high coverage in routine immunization. National EPI programs should use experiences gained from the switch to develop evidence-based and context-specific communication and crisis management strategies for routine immunization.

## CONCLUSIONS

The switch in South-East Asia was a unique and truly unprecedented event in public health history. Due to the emerging epidemiological risks of type-2 poliovirus, tOPV withdrawal had to happen almost simultaneously in all countries in the region, and around the world. Because the switch was part of the polio eradication Endgame Plan, the process had particular impetus and urgency. Moreover, the switch brought additional resources to routine immunization and a renewed global focus to make it a success. Ultimately, however, the success of the switch implementation process in the SEAR was due in large part to the strength of the commitment in the countries to the goal of polio eradication and effective routine immunization. A very strong partnership formed between the WHO and UNICEF regional offices and in-country stakeholders, which enabled a comprehensive approach to monitoring, analysis, and technical support to countries for the switch. 

There are several important technical lessons that immunization programs in the region can take from the switch that relate to vaccine stock management, health worker training, communication and crisis management, and monitoring. In addition, the process for the delivery of the switch in the region may also be transferrable to other activities in routine immunization. There are a number of forthcoming opportunities in the region for immunization programs to use a similar approach to that used for the OPV switch. This includes an exercise (almost identical to the 2016 switch) in 2019 when the world takes the next step toward polio eradication by completely withdrawing OPV use. Moreover, several countries in South- East Asia are preparing for the introduction of rotavirus vaccine and other vaccines, changes in immunization schedules, and strengthening of immunization systems. The lessons from the OPV switch should be studied in detail as part of these activities.

In conclusion, the innovative approach taken in the SEAR demonstrated the value of extensive engagement of national immunization programs based on risk identification, monitoring and strategic planning, and implementation. This engagement strengthened the existing commitments to polio eradication and provided a particular focus on vaccine supply, logistics, communication, health worker training, and monitoring for routine immunization.

## Notes

***Acknowledgments.*** We thank the senior management and staff of the regional and country offices of the WHO and UNICEF, especially Dr Mandeep Rathee, Uttara Agarwal, Tika Sedai, Dr Aarti Garg, Seema Kapoor, and Deepak Dhongde for providing organizational and data support to the switch process in the region. We also thank all health managers and frontline workers of the Ministries of Health of all SEAR countries for their active participation, coordination, and leadership in the planning, preparation, implementation, and monitoring of the switch in their respective countries.

***Disclaimer.*** The observations and conclusions in this report are those of the authors and do not necessarily represent the official position of the WHO and UNICEF.

***Supplement sponsorship***. This work is part of a supplement coordinated by the Task Force for Global Health with funding provided by The Bill and Melinda Gates Foundation and the Centers for Disease Control and Prevention.

***Potential conflicts of interest.*** All authors: No reported conflicts. All authors have submitted the ICMJE Form for Disclosure of Potential Conflicts of Interest. Conflicts that the editors consider relevant to the content of the manuscript have been disclosed.

## References

[1] Hasman A Raaijmakers HC Noble DJ Inactivated polio vaccine launch in Nepal: a public health milestone. Lancet Glob Health 2014; 2:e627–8. 25442682 10.1016/S2214-109X(14)70324-9

[2] World Health Organization. Polio vaccines—WHO position paper—March 2016. Wkly Epidemiol Rec 2016; 91:145–68.27039410

[3] World Health Organization. Report of the South-East Asia Regional Consultation on the Polio Endgame Strategy. Bangkok, Thailand: WHO Regional office for South-East Asia, 2012.

[4] Meeting of the Strategic Advisory Group of Experts on immunization, October 2015—conclusions and recommendations. Wkly Epidemiol Rec 2015; 90:681–700. 26685390

[5] World Health Organization. WHO Global Action Plan to minimize poliovirus facility-associated risk after type-specific eradication of wild polioviruses and sequential cessation of oral polio vaccine use (GAP III) http://polioeradication.org/wp-content/uploads/2016/12/GAPIII_2014.pdf Accessed 26 October, 2016.

[6] Anand A Zaman K Estivariz CF Early priming with inactivated poliovirus vaccine (IPV) and intradermal fractional dose IPV administered by a microneedle device: a randomized controlled trial. Vaccine 2015; 33:6816–22. 26476367 10.1016/j.vaccine.2015.09.039 PMC10423714

[7] World Health Organization. Report of the WHO SEAR ITAG. June 2016 http://www.who.int/immunization/sage/meetings/2016/october/9_7th_Meeting_of_SEAR-ITAG_Final_version_15092016_updated_Sept_26_CF.pdf?ua=1 Accessed 26 October, 1996..

